# Supplementary material for: Modelling motility of Trypanosoma brucei
Source: PLoS Comput Biol. 2025 May 21;21(5):e1013111. doi: 10.1371/journal.pcbi.1013111 (PMC12136433; doi:10.1371/journal.pcbi.1013111)
Supplement: S1 Appendix — (PDF) [file pcbi.1013111.s001.pdf]

S1 Appendix. Bending stiffness of the flagellum

To measure bending stiffness K of the flagellum, we fix several first segments of a straight flagellum without the body and apply a load P on the last segment of the free flagellum side. Deflection $w(x)$ in the direction of the force from the Euler–Bernoulli beam theory [1] is given by

$$w(x) = \frac{Px^2(3L_{flag} - x)}{6K}, \tag{1}$$

where K is the bending rigidity. Then, K is computed from $w_{max} = w(x = L_{flag})$ as $K = PL_{flag}^3/(3w_{max})$.

References

[1] Gere JM, Timoshenko S. Mechanics of materials. Boston: PWS Publishing Company; 1997.
