# Supplementary material for: Modelling motility of Trypanosoma brucei
Source: PLoS Comput Biol. 2025 May 21;21(5):e1013111. doi: 10.1371/journal.pcbi.1013111 (PMC12136433; doi:10.1371/journal.pcbi.1013111)
Supplement: S2 Appendix — (PDF) [file pcbi.1013111.s002.pdf]

S2 Appendix. Fluid model

Fluid environment surrounding the *T. brucei* model is simulated using the smoothed dissipative particle dynamics (SDPD) method [1–3], a mesoscale hydrodynamics simulation technique derived through a particle-based Lagrangian discretization of the Navier-Stokes equation. SDPD particles represent small fluid volumes, and the method produces consistent thermal fluctuations [4]. The total force $\mathbf{f}_i$ acting on particle i is the sum of all interactions with neighboring particles within a cutoff radius r_c

$$\mathbf{f}_i = \sum_{j \neq i} \mathbf{F}_{ij}^C + \mathbf{F}^T + \mathbf{F}^R + \tilde{\mathbf{F}}_{ij}, \tag{1}$$

where $\mathbf{F}_{ij}^C$ is the conservative force, $\mathbf{F}^T$ and $\mathbf{F}^R$ are the translational and rotational dissipative forces, and $\tilde{\mathbf{F}}_{ij}$ is the random force. These forces are expressed as follows

$$\begin{aligned}
\mathbf{F}_{ij}^C &= \left(\frac{p_i}{\rho_i^2} + \frac{p_j}{\rho_j^2}\right) F_{ij} \mathbf{r}_{ij}, \\
\mathbf{F}_{ij}^T &= -\gamma_{ij} \left(\mathbf{v}_{ij} + \hat{\mathbf{e}}_{ij} \left(\hat{\mathbf{e}}_{ij} \cdot \mathbf{v}_{ij}\right)\right), \\
\mathbf{F}_{ij}^R &= -\gamma_{ij} \frac{\mathbf{r}_{ij}}{2} \times \left(\mathbf{s}_i + \mathbf{s}_j\right), \\
\tilde{\mathbf{F}}_{ij} &= \zeta_{ij} \left(d\overline{\boldsymbol{\mathcal{W}}}_{ij}^S + \frac{1}{3}\mathrm{tr}[d\boldsymbol{\mathcal{W}}_{ij}]\mathbb{1}\right) \cdot \frac{\hat{\mathbf{e}}_{ij}}{\Delta t},
\end{aligned} \tag{2}$$

where p_i is the pressure at particle i, ρ_i is the particle density, $\mathbf{r}_{ij} = \mathbf{r}_i - \mathbf{r}_j$, $\hat{\mathbf{e}}_{ij} = (\mathbf{r}_i - \mathbf{r}_j)/|\mathbf{r}_{ij}|$, $\mathbf{v}_{ij} = \mathbf{v}_i - \mathbf{v}_j$, $\mathbf{s}_i$ is the angular velocity (or spin), and the function F_{ij} is derived from the kernel function W_{ij} as $\nabla_i W_{ij} = -\mathbf{r}_{ij} F_{ij}$. The kernel function is defined to be the Lucy function [5] defined as

$$W_{ij} = \frac{105}{16\pi r_c^3} \left(1 + 3\frac{r}{r_c}\right) \left(1 - \frac{r}{r_c}\right)^3, \tag{3}$$

and the particle density is calculated as

$$\rho_i = \sum_j m_j W_{ij}, \tag{4}$$

where m_j is the mass of particle j. The friction coefficient ζ_{ij} is related to fluid dynamic viscosity η and the random force coefficient σ_{ij} as

$$\gamma_{ij} = \frac{20\eta}{7} \frac{F_{ij}}{\rho_i \rho_j}, \quad \zeta_{ij} = 2\sqrt{k_B T \gamma_{ij}}, \tag{5}$$

where $k_B T$ is the energy unit. The term $\mathrm{tr}[d\boldsymbol{\mathcal{W}}_{ij}]$ represents the trace of a matrix of independent Wiener increments, while $d\overline{\boldsymbol{\mathcal{W}}}_{ij}^S = d\boldsymbol{\mathcal{W}}_{ij}^S - \mathrm{tr}[d\boldsymbol{\mathcal{W}}_{ij}]\mathbb{1}/3$ is its traceless symmetric part. Δt is the time step. The equation of state for the pressure is chosen as

$$p = p_0 \left(\frac{\rho}{\rho_0}\right)^\alpha - p_l, \tag{6}$$

where ρ_0 is the nominal fluid density, and p_0, α, and p_l are the parameters which control fluid compressibility with the speed of sound $c_s^2 = p_0\alpha/\rho_0$.

The trypanosome model is immersed into the SDPD fluid, such that particles within the trypanosome structure serve as discretization points for fluid flow at the moving parasite surface. In this way, no-slip boundary conditions at the parasite surface are automatically satisfied. Note that SDPD particles can cross the membrane so that the SDPD fluid fills both inside and outside the parasite body. The simulation system is integrated using the velocity-Verlet algorithm [6]. The implementation of all methods and models is performed within the parallel software package LAMMPS [7].

In all simulations, the SDPD fluid parameters are $\rho_0 = 2.7 \times 10^5/L_{tryp}^3$, $p_0 = 2.7 \times 10^8 k_BT/L_{tryp}^3$, $p_l = 2.65 \times 10^8 k_BT/L_{tryp}^3$, $\alpha = 7$, and $r_c = 0.04L_{tryp}$. The mass of fluid particles is set to $m_f = 5.625k_BT/(L_{tryp}f)^2$, while the mass of trypanosome particles is $m_{tryp} = 10m_f$, which allows us to use a time step of $\Delta t = 3.125 \times 10^{-5}/f$. We have also performed several simulation runs using $m_{tryp} = m_f$ with a significantly reduced time step to verify that the trypanosome swimming characteristics are not affected by possible inertial effects.

References

[1] Español P, Revenga M. Smoothed dissipative particle dynamics. Phys Rev E. 2003;67:026705.

[2] Müller K, Fedosov DA, Gompper G. Smoothed dissipative particle dynamics with angular momentum conservation. J Comput Phys. 2015;281:301–315.

[3] Alizadehrad D, Fedosov DA. Static and dynamic properties of smoothed dissipative particle dynamics. J Comput Phys. 2018;356:303–318.

[4] Vázquez-Quesada A, Ellero M, Español P. Consistent scaling of thermal fluctuations in smoothed dissipative particle dynamics. J Chem Phys. 2009;130:034901.

[5] Lucy LB. A numerical approach to the testing the fission hypothesis. Astronom J. 1977;82:1013–1024.

[6] Allen MP, Tildesley DJ. Computer simulation of liquids. New York: Clarendon Press; 1991.

[7] Thompson AP, Aktulga HM, Berger R, Bolintineanu DS, Brown WM, Crozier PS, et al. LAMMPS - a flexible simulation tool for particle-based materials modeling at the atomic, meso, and continuum scales. Comp Phys Comm. 2022;271:108171.
