# Supplementary material for: Modelling motility of Trypanosoma brucei
Source: PLoS Comput Biol. 2025 May 21;21(5):e1013111. doi: 10.1371/journal.pcbi.1013111 (PMC12136433; doi:10.1371/journal.pcbi.1013111)
Supplement: Attachment — Submitted filename: response_referees_v1.pdf [file pcbi.1013111.s012.pdf]

Response to the reviewers' comments

Manuscript ID: PCOMPBIOL-D-24-02058

Title: Modelling Motility of *Trypanosoma brucei*

Authors: Florian A. Overberg, Narges Jamshidi Khameneh, Timothy Krüger, Markus Engstler, Gerhard Gompper, Dmitry A. Fedosov

Dear Editor,

Thank you very much for sending us the reviewers' comments on our manuscript. We would like to thank the reviewers for their careful reading of our manuscript and helpful comments. We have addressed all raised points with our responses below. Changes in the revised manuscript are marked in red.

We hope that the manuscript is now suitable for publication in PLOS Computational Biology.

Sincerely yours,

Florian A. Overberg, Narges Jamshidi Khameneh, Timothy Krüger, Markus Engstler, Gerhard Gompper, Dmitry A. Fedosov

Response to the Reviewer 1

We thank the reviewer for the helpful suggestions.

Some suggestions and recommendations:

1. The title "Modelling Trypanosome Motility" suggests a generic model, however, the simulations are based on the analysis of T. brucei motility. It is important to specify how the model could be adapted for different Trypanosoma species, as morphological variations among species may influence motility.

We have changed the title to 'Modelling Motility of *Trypanosoma brucei*'. Possible adaptations of the model, which are straightforward to do, are to change the parasite geometry and flagellum beating characteristics. We do mention these model adaptations in the discussion section, which can represent different morphological forms of trypanosomes. Therefore, we think that our model is more general than for *T. brucei* only.

2. The authors state that the developed model realistically mimics the swimming behavior of trypanosomes; however, the model was created without considering the presence of cells in the medium. It is known that certain species, e.g., T. cruzi trypomastigotes, can detect the presence of mammalian cells and alter their motility patterns. Therefore, it would be valuable for the authors to include a discussion on the limitations of their model and clarify that it is initially applicable to scenarios where the parasite swims freely in the fluid. Additionally, they should discuss how interactions with the cellular environment affect motility and the model's parameters.

An active change of the motility pattern by the parasite in reaction to environmental conditions is currently not a part of our model, though some aspects can certainly be included. In response to the comment of the referee, we have expanded the discussion of potential effects an environment can have on the trypanosome motility.

3. The proposed modeling approach may be challenging due to the parameters involved. Using artificial intelligence (AI) techniques based on experimental data would allow for more robust parameter adjustments, optimizing the model and improving its adaptability to different species. One suggestion would be for the authors to dedicate a step-by-step section explaining how this could be achieved.

We are aware that the usage of AI for the extraction of parameters from experimental data is an active research field. However, there is a large number of parameters necessary to fully capture trypanosome motility, and there are not enough data available for a data-driven AI approach so far. On the other hand, experimental measurements have sufficient precision for the numerical modelling presented here.

Response to the Reviewer 2

This paper describes a three dimensional simulation of a Trypanosoma brucei cell, used to simulate how flagellum beating leads to swimming behaviour.

It is similar but represents an improvement on the previous model two of the authors were previously involved with (Alizadehrad et al 2015) by being a higher resolution simulation, and avoiding some key failures to accurately capture the cell shape. Specifically, having the flagellum run on the outside of the approximately helical cell shape, and having a beat amplitude comparable to real swimming cells. But, it also has some of the same failings: Specifically, not trying to consider the likely highly anisotropic mechanical properties of the cell body cytoskeleton. Additionally, it anchors beat plane orientation to the cell body, and appears to have incorrectly parameterised the trypanosome width.

Overall, the paper is clearly written, from my more biological background appears methodologically sound, and draws reasonable conclusions from the simulations and data presented.

We thank the reviewer for the thorough reading and detailed comments.

However, I would like to highlight two major limitations for two of the major conclusions: Regarding contribution of cell body deformability to cell movement: 1) Not analysing the anisotropic cell body cytoskeleton misses one of the very characteristic properties of trypanosome cell organisation. Regarding the flagellum beat plane orientation relative to the cell body: 2) Analysing a flagellum beating with a fixed beating plane relative to the cell body likely does not reflect the biomechanical properties of the actual flagellum-cell body connection.

We will address these issues in detailed responses below and hope to convince the reviewer that while the criticism raises fair points, we do not expect them to qualitatively change the results.

Major comments and queries:

Methodology The cell body is simulated as an elastic mesh. The overall geometry is broadly reasonable, however seems to give a very wide cell, despite Rmax being set to 1.5 um. See, for example, the side by side comparison in Figure 3. Based on the scale bar in Fig 3, the actual diameter is perhaps 6 um instead of the target 3 um, unless there are other scaling issues. I imagine that scaling the cell body diameter to correct this would not fundamentally change the results, but would shift several plots - this needs to be carefully checked.

In simulations, the parasite length is $L_{tryp} = 24\ \mu m$ and the maximum diameter is 3 μm

or $R_{max} = 1.5\ \mu m$, as we illustrate in Fig. 1(a). Regarding Fig. 3, there is a scaling issue. The scale bar is only relevant for the experimental images. We have re-scaled simulation images to avoid confusion.

The isotropic triangulation and harmonic spring potential used to model the cell surface cytoskeleton doesn't capture the highly anisotropic organisation of the parasite cytoskeleton (eg. Sinclair and de Graffenried 2020). The cell surface is underlain by a parallel array of microtubules maintained at approximately constant spacing - microtubules are relatively stiff and incompressible, and the consistent spacing suggests a microtubule-microtubule distance constraint. This likely gives anisotropic mechanical property of the cell surface, which is not captured. I can find no mention of the sub-pellicular microtubules of the cytoskeleton, and no citation of corresponding literature. There are relevant recent pieces of work from the de Graffenried, He, Vaughan and Varga research groups, older work from the Gull group, and original electron microscopy prominently from Keith Vickerman. At minimum, this needs to be better explained and discussed. And would it be possible to test an anisotropic situation? There is sub-pellicular microtubule arrangement data available for T. brucei procyclic forms from expansion microscopy (Gorilak et al 2021) and Leishmania promastigotes from electron tomography (Hair et al 2024) as guides.

This is an important point. Indeed, trypanosomes have a microtubule corset running from the posterior to the anterior end, which is not implemented in our model. The incorporation of this structure in the model may very well alter the body elasticity and deformation due to flagellar actuation. This could also affect the results quantitatively. However, qualitatively, our results indicate that a stiffer body (or less deformation) can significantly reduce parasite rotation, but does not strongly affect the swimming velocity. We expect a similar effect of an explicit corset on the swimming properties.

Note that from the mechanical point of view, the situation is likely much more complex. As the parasite swims, the whole body is bent, and not only the microtubule corset locally. To estimate bending rigidity K_{body}, we can approximate the body as a cylinder. For a thin hollow cylinder, $K_{body} \sim E_s R^3$, where E_s is the surface Young's (or stretching) modulus and R is the cylinder radius. This means that bending rigidity of the whole body in our model is not isotropic due to varying radius, despite isotropic elasticity modulus E_s. For a filled cylinder, $K_{body} \sim E_b R^4$, where E_b is the bulk Young's modulus. In fact, trypanosome body can be considered as a composite material, with a thin corset (a hollow cylindrical structure) and bulk cytoskeleton (a filled cylindrical structure), and it is not clear what would be the dominant contribution. In addition, both elastic properties (E_s and E_b) might be non-isotropic. Bending rigidity of microtubules may also contribute to K_{body}, but it must have at most secondary effect. When we change the membrane bending rigidity in our model by two orders of magnitude, we see very little effect on trypanosome swimming, because membrane stretching elasticity E_s dominates K_{body}. While we think that it is worthwhile to study the effect of stiff filaments running along the body axis, we

want to emphasize that this is not a trivial task and exceeds the scope of our work.

We have added a discussion of the role of microtubules, the fact that we do not include them in the current model, and that their incorporation is an interesting aspect for future model development.

The energy potential for control of surface area is surely redundant, given the triangulated springs of the cell surface? Is there a way to probe its contribution, which would add confidence that the modelling of the cell surface as isotropic springs is providing the expected constraint, rather than this additional surface area constraint? A simple way to test this may be to simply remove the surface area constraint and see similar behaviour.

Both springs and the area constraint contribute to area control, since the area-compression modulus is equal to $\sqrt{3}k_{s,b}/2 + k_{A,glob} + k_{A,loc}$, where $k_{s,b}$ is the spring stiffness. We have tested changes in local and global area conservation coefficients during the development of the model, and their effect on the swimming characteristics was indeed weak ($< 10\%$). Note that the area conservation constraints are somewhat important for low body elasticity (i.e., low $k_{s,b}$). Furthermore, the local area constraint stabilizes simulations (at least for low values of $k_{s,b}$), since it does not allow very strong local compression and stretching of springs. We have commented on this in the revised manuscript.

Figure 5/Body and flagellum deformability My concerns about 1) cell width scaling and 2) failure to capture the anisotropic parasite cytoskeleton mechanical properties limit the accuracy of any conclusions drawn here.

Both points have been addressed in comments above. Regarding the first point, maximum diameter of the trypanosome is 3 μm, and we have re-scaled the simulation picture to avoid confusion. For the second point, even though the explicit effect of microtubules in trypanosomes is not covered by our model, the conclusions drawn are very reasonable, at least for the qualitative dependence of trypanosome swimming characteristics on body elasticity.

Figure 7 and 8/Tangential versus normal beating plane of the flagellum. It is stated that "The orientation of the flagellum beating with respect to the body surface is very difficult to extract experimentally", but this is not true. Perhaps, difficult to determine from high speed videos. But flagellum bending occurs perpendicular to the midline between the axoneme central pair, and the orientation of the central pair is trivial to observe from electron microscopy. Similarly, the attachment plaque of the flagellum attachment zone, and its position relative to the axoneme, is trivial to observe from electron microscopy.

The criticised sentence is indeed not true for some given positions along the cell body, so that we have removed it. What we tried to say is that the beating orientation along the entire length of the body has not been determined and thus is not known, despite the fact

that the orientation of the flagellum is trivial to observe from single electron micrographs.

The model uses a fixed beat orientation to the cell body, achieved by anchoring both of either the oscillating or non-oscillating filaments as part of the cell body mesh. In biological terms, this corresponds to assigning the anchoring of the flagellum to the cell body via the flagellum attachment zone to a fixed doublet of the axoneme, and thus fixing the orientation of attachment relative to the plane of flagellum bending. In practice, the FAZ position around the axoneme is variable - Wheeler et al 2013 Figure 3 conveniently has several examples, where the point of attachment varies between doublet 6 and 8, corresponding to a 90 degree rotation of the axoneme, and thus rotation of bending orientation, relative to the cell body. From this quick observation, it is evident that the axoneme is free to rotate such that the beat plane can be either perpendicular or normal to the attachment to the cell body.

The constant beating-plane orientation is a simplification that deviates from the more complex biological system. We respectfully disagree with the conclusion that different flagellum orientations at different positions suggest that the flagellum is free to rotate and change its beating plane. The attachment of the flagellum seems to be stable at a given position (Sunter et al, 2015), but this stable orientation can change from perpendicular to normal along the body. Furthermore, the exact binding mechanism between FAZ and flagellum is not known (de Liz et al., 2023). It is possible that the "staple" complexes are somewhat flexible, so that they allow a free 90° rotation, but observations of flagellar behaviour do not suggest this kind of free flapping along the flagellum length.

We agree that the previous experimental work, e.g. Wheeler et al., 2013, indicates that the beating-plane orientation varies along the cell. In fact, a somewhat similar behavior has been observed for sperm, where the beating plane is not only changing at a fixed point along the flagellum but actually at a point which travels from the head to the tail part of the flagellum together with the wave, see Gong et al., Eur. Phys. J. E 44, 87 (2021). Both types of variation of the beating plane can be incorporated in our model. However, this needs experimental input about the biological system. Therefore, we restrict our study here to a constant beating plane.

This is a major limitation for the results illustrated in Figure 8. The result of the simulation is interesting, but does not reflect the reality that the axoneme actually appears able to rotate by 90 degrees relative to the FAZ. It would be much more informative to have the axoneme able to freely rotate relative to the cell body, then ask what orientation of beat it then prefers to attain. This could be achieved by changing anchoring of the axoneme to the cell to instead use an axoneme midline of vertices, which are then attached to the four axoneme filaments by radial connections. At minimum, this key limitation needs to be acknowledged.

We agree that the free orientation of the axoneme is a very interesting aspect of trypa-

nosome motility. We think that our current results with two different beating planes can already provide some insight into this issue, as we find that the tangential beating deforms the body much less, and therefore also generates less dissipation. We therefore conjecture that this will be the preferred orientation if the beating plane adjusts itself. We now mention the study of a freely adjusting beat plane as possible direction of future research in the conclusion.

Minor points

The cell volume constraint is important. It is mentioned qualitatively that volume preservation vs. considering a viscoelastic cytoplasm is likely to have a small effect, and I agree that the difference likely is small, but a quantitative statement would be nice if possible.

We employ frictional coupling of the parasite to a background fluid. Therefore, fluid particles fill the whole computational domain, and are also present inside the parasite body. Note that fluid particles can cross the membrane, so that the internal fluid does not enforce a desired volume, for which the volume constraint is essential. Our tests during the development of the model showed that without volume constraint, body deformation might be sometimes extreme (membrane on one side of the cross-section nearly touches the membrane on the other side). Again, a more realistic representation of internal mechanical properties may affect body deformation, but it a complex task, as we do not know enough about the mechanics of internal structures. We mention the importance of volume constraint in the revised manuscript.

"What are the available experimental observations" which define the path of attachment of the flagellum to the cell body. I am more familiar with the procyclic life cycle stage morphology, and the attachment path does not match my expectations from that life stage. This work is, of course, describing the bloodstream form instead - but this raises a minor concern about the accuracy of this aspect of the model, and it is not a tested aspect of the model.

This information mainly comes from the experimental coauthors at the University of Würzburg. We have improved such statements and included references where possible.

Was the viscosity of methylcellulose-HMI9 measured? If so how, and if not, how was this viscosity inferred?

The viscosity was measured and provided by the supplier.

Please carefully make it clear what is experimental data vs. simulation, as a prominent example, Figure 2 legend presents simulation as "Swimming characteristics of a trypanosome".

Thanks for pointing out this ommission. We have now clarified the source of the data in the figure captions.

”Since the trypanosome flagellum is accompanied by a paraflagellar rod running in parallel, we chose a K value that represents a relatively stiff flagellum.” Perhaps true, but worth expanding to note that the PFR runs along doublets 4 to 6, it is stretched and squashed by beating (even sitting on the inside of the more tightly curved bend of the asymmetric Leishmania beat, Wang et al 2020). You should also consider the biomechanical spring model of PFR function (Hughes et al 2012) - while I think this is unlikely to be correct, it is worth explicit mention.

We have slightly expanded the discussion and mention the suggested references in the revised manuscript.

Relating to Figure 6, is there any biological basis for differences in the proximal flagellum which might confer a larger or smaller actuation force? Eg. differences in axoneme composition.

We are not aware of any direct evidence. However, our experimental observations seem to indicate that there are differences in amplitudes, depending on which part of the flagellum is considered. In simulations, this can be reproduced by altering the local wavelength, which we present in Fig. 6. How this is realized microscopically, we cannot say exactly. One component will most likely be the body rigidity, which damps the bending wave and hence the wave amplitude stronger at regions with higher body radius.

There are several places where “in agreement with experimental observations” is stated with no reference. I realise that these tend to be quite general statements, but some reference really is required, even if just a paper with a good illustrative video rather than specifically talking about that point.

We have added references where possible or modified the corresponding sentences.

I am coming from a biological background, and I acknowledge some bias from this, but there was limited citation of trypanosome cell biology research groups with interests in motility and associated cell morphology - particularly He, Wheeler and Hill - and a tendency for self-citation of work from the Engstler group.

We have added a few references to our reference list to make it more balanced.

It is stated that ”the unactuated shape of the trypanosome flagellum is not known”. This is true, but really is very likely to be straight due to the rigidity of the microtubule-based structure. It would also be easy to test experimentally by lysis of trypanosomes and treatment with Ca2+ to depolymerise the sub-pellicular microtubules.

We agree that from the microtubule-based structure, the flagellum can be expected to have a straight minimum-energy shape. A possible argument for a bent shape is that the PFR or the cell body may have a bent shape and thus, force the flagellum to some bent shape. We would be glad to learn more about it, when such experiments are performed in the future.

Response to the Reviewer 3

This paper describes a numerical model of T. brucei, a eukaryotic parasite of humans, livestock and other animals. The authors build a simulated cell body using a mesh of particles and add a simplified model flagellum to the cell body, composed of four filaments, an opposing pair of which are actuated to produce a flagellar beat. The authors investigate the shape of the flagellar beat (output wavelength, beat plane) as a function of various external and internal quantities including bending stiffness of the flagellum, amplitude of the input waveform etc. They conclude by outlining some future directions of research.

This is a nicely executed technical study on an important organism. I'm not too familiar with the numerical details of the simulations, but I have seen the SDPD before and it seems to be a well-established method. The parameter space is large but the authors make reasonable choices about which regimes to investigate, to maintain biological relevance. The results look sound to me, and this will be a useful tool in future studies, but I have a few queries around the application of the method and its interpretation.

We thank the reviewer for the positive feedback and helpful comments.

1. The model includes harmonic potentials between 'atoms' within the cell body and within the flagellum; this implies that there is elasticity in the cell body but I can't see that there is a viscosity. Presumably, given the highly heterogeneous composition of cell membrane and cytoplasm, the body will contribute a viscous as well as elastic response, and that this viscous response (as opposed to interactions with the external fluid) may dominate the dissipation. Can the authors comment on this? Viscosity might lead to consequences including a phase lag between actuation and response that significantly alters the beat plane, body shape etc. It may simply be that incorporating a viscoelastic response in the cell body would make the computational requirements a lot more challenging - in which case, there is little choice but to leave it out.

We employ frictional coupling of the parasite to a background fluid. Therefore, fluid particles fill the whole computational domain, and are present both inside and outside of the trypanosome body, contributing to both internal and external viscous response in addition to the elasticity. Of course, it is possible that for real parasites, the fluid viscosity and dissipation inside are larger than what we have in the current model. A larger viscosity inside would lead to less deformation of the body for the same actuation strength due to larger dissipation. This is similar to a stiffer body (i.e., less deformation), and our results indicate that a stiffer body can significantly reduce parasite rotation, but does not strongly affect the swimming velocity. We added a comment about this in the discussion.

2. The paraflagellar rod is mentioned in the introduction but then omitted in the later discussions. This structure is located next to the axoneme in the real cell and presumably

lends an anisotropic bending stiffness to the flagellum in the real-world case. In fact, it seems that the PFR varies in length throughout the life cycle, both in absolute terms and relative to the rest of the flagellum (e.g. Bastin, Mol. Cell. Biol. 19(12) p.8191, 1999). Could the authors comment in its potential relevance in the discussion/conclusion section?

We agree that the PFR must affect trypanosome beating dynamics, though we did not explicitly include it in the model. If the PFR affects local bending stiffness, then the flagellar beating might locally change. This can of course affect the swimming properties quantitatively, but we expect no qualitative changes in the swimming behavior. We have extended the discussion to mention a possible importance of the PFR.

3. In the discussion, the authors state that their simulations "confirm the hypothesis that trypanosome propulsion is primarily generated by the anterior part of the flagellum". The simulations are consistent with this hypothesis, but 'confirm' is too strong. The parameters were chosen to mimic real life, so claims of 'confirmation' appear to be circular logic. I would suggest softening the claim.

We adapted the sentence, where we say that it supports the hypothesis.

4. When observing Trypanosomes under the microscope, I've been surprised by cells that appear to move vigorously, but make little forward progress. This isn't something for the current manuscript, but I wonder if this numerical model can help form mechanical hypotheses for why the cells exhibit this behaviour, and whether it's some sort of adaptation for another environment with different mechanical properties.

We have shown that forward and backward flagellar waves can cancel each other and cause tumbling (Heddergott et al., 2012). This can be implemented and tested in future simulations. We have also shown that the persistent forward motion or the reversal of beating can be influenced by the environment in the same publication.

Minor issue
1. Could the authors put a dashed line with a slope of Sp^{-4} on Figure 4, as a guide to the eye?

We have changed the figure so that the line Sp^{-4} is dashed and clearly visible.
