# Supplementary material for: Modelling motility of Trypanosoma brucei
Source: PLoS Comput Biol. 2025 May 21;21(5):e1013111. doi: 10.1371/journal.pcbi.1013111 (PMC12136433; doi:10.1371/journal.pcbi.1013111)
Supplement: Attachment — Submitted filename: response_referees2.pdf [file pcbi.1013111.s013.pdf]

Response to the reviewers' comments

Manuscript ID: PCOMPBIOL-D-24-02058R1

Title: Modelling Motility of *Trypanosoma brucei*

Authors: Florian A. Overberg, Narges Jamshidi Khameneh, Timothy Krüger, Markus Engstler, Gerhard Gompper, Dmitry A. Fedosov

Dear Editor,

Thank you very much for sending us the reviewers' comments on our revised manuscript. We are happy that most concerns were successfully resolved. We have addressed now all remaining points with our responses below. Changes in the revised manuscript are marked in red.

We hope that the manuscript is now suitable for publication in PLOS Computational Biology.

Sincerely yours,

Florian A. Overberg, Narges Jamshidi Khameneh, Timothy Krüger, Markus Engstler, Gerhard Gompper, Dmitry A. Fedosov

Response to the Reviewer 2

Thank you for your comprehensive efforts to address my comments. I find the text markedly improved, particularly regarding discussions of some limitations/simplifications, referencing of the wider biological literature to place comments in better context, and the clarify of explanation of some methodological areas. Arguments against further simulation to test, for example, PFR stiffness or cortical cytoskeleton anisotropy are reasonable given lack of experimental data for the corresponding physical properties - although perhaps it would be nice for simulation to lead experiments here.

We are happy to see that the referee finds our revised manuscript significantly improved.

However, my key concern of cell size has not been addressed. The authors assure that the size of the model trypanosome matches reality, and have adjusted scale of some figure panels. However this does not address the root concern here.

We apologize for this misunderstanding. Clearly, in our simulation model we had to make a choice for the trypanosome geometry, which is within experimentally measured variability of parasite geometries. Therefore, the model should of course not be considered as an exact mimic of real parasite, but as a good approximation of the parasite geometry. The shape can be adapted if necessary, but we do not expect any qualitative differences in parasite behavior as long as the adaptation is moderate.

I measured the width/length ratio from the model and microscopy images in Figure 3. I get $R_{max} \sim 0.08 L_{tryp}$ for the model, larger than the stated 0.0625 from Table 1 presumably due to some out-of-plane bending. I get $R_{max} \sim 0.04 L_{tryp}$ for the microscopy images. A similar near two-fold mismatch in cell width can be seen by just overlaying the model and microscopy images, having scaled to roughly match length. I don't seem to be able to attach an image to illustrate this, so please do try the overlay and observe. Impact of cell width on the simulation is not tested, so critical to observe and set it correctly. The width of the cell stated in Table 1 is $R_{max} = 1.5 \mu m$. Why was this value selected? What references or measurements have guided this? I am afraid that statements like 'the modelled body shape closely captures geometrical features of real T. brucei cells' cannot be true when simple observation suggests a two-fold difference in the width/length ratio.

In the model, $R_{max} = 1.5 \mu m$ (total thickness of $3 \mu m$) is selected based on the previous trypanosome model by Alizadehrad et al. PLoS Comput. Biol. 11:e1003967 (2015). Experimental measurements from the group of M. Engstler indicate that the thickness of *T. brucei* normally lies in the range $2 - 3 \mu m$, so that our model is near the upper limit. Note that during beating, the body deforms and its thickness can change. Our simulation results indicate that the maximum thickness slightly increases (up to about $3.5 \mu m$) due to body deformation. The experimental example in Fig. 3 is a trypanosome with an apparent thickness of about $2.4 \mu m$; it is unclear to which extent this value is affected by cell

deformation. Thus, we completely agree with the reviewer that the experimental example of trypanosome displayed in Fig. 3 is thinner than the modeled trypanosome.

We do not expect that a moderate change in thickness will qualitatively change the simulated behavior. Changes in thickness will affect swimming resistance and body deformation. A thinner parasite should have less drag and thus swim slightly faster. The effect of body deformation on swimming has been studied in the manuscript by changing body rigidity. In response to the reviewer, we have clarified our choice of trypanosome thickness and modified the statement mentioned by the reviewer.
